# A Study of the "End Result" System

**Published:** 1918-03-23

**Authors:** 


### THE FINANCE OF SURGERY.

### A Study of the "End Result" System.

The problems of hospital management have been approached from many standpoints, and since the days when institutional affairs were first treated on as scientific a basis as, say, international finance, vast strides have undoubtedly been made in placing them upon a sure foundation. But certainly the author of the pamphlet now before us* has struck out for himself what can truthfully be described as an entirely new line. The surgeon and administrator for some years of a small privately owned nursing home in Boston, Mass., he has worked out upon an exact basis the profit and loss incurred by receiving patients at an inclusive charge of 100 dollars for the first three weeks of their stay, with a subsequent payment of 25 dollars for each successive week that they remain. This fee includes the operation, subsequent nursing and maintenance, and the supply of any drugs or, to a limited degree, appliances that may be needed during the period of their recovery. In addition he appends an abstract of 270 cases operated upon or treated by him during the four years that the experiment has continued, with notes indicating (in an extraordinarily frank manner) the success or failure of the treatment adopted and the probable causes therefor. In this lies the innovation. For no hospital or institution for the treatment of the sick, so far as we are aware, has ever before published such a series of cases. Tables there are galore of selected cases on every known disease, exemplifying the results, brilliant or otherwise, of a particular method of treatment, but no hospital has previously had the courage to publish the results of its treatment upon each individual case admitted thereto, with a concise opinion of the surgeon in charge of the result of the treatment adopted, whether correct or faulty, either from errors in diagnosis, technique, or surgical judgment.

### Surgical Service as a Commercial Product.

The doctor is not content with a mere dry-as-dust summary in results, but in an extremely witty foreword exposes his system of medical ethics. "This hospital," he says, "has for sale a product of the standard found on the first twenty-six pages. It aims to be a 100-dollar hospital with a 100-dollar surgeon." Such a frank avowal lays itself open to all the conventional charges of commercialism and self-advertisement. But if we probe a little deeper and examine without prejudice the aims of what its originator calls the "End Result" system, we may find ourselves convinced that this notion of surgical and possibly medical service as a commercial product in the strict sense of the word, is by no means inimical to the best interests of the profession, and of the lay public who utilise its services. The idea is a simple one, but it has until now been practically neglected in all charitable hospitals, and entirely so in the private home. It is simply to follow the natural series of questions which anyone asks in an individual case: What was the matter? Did they find it out beforehand? Did the patient get entirely well? If not—why not? Was it the fault of the surgeon, the disease, or the patient? What can we do to prevent similar failures in the future?

The author believes that the general acceptance of this system of hospital organisation, based on a truthful record of the answers to these questions, means the beginning of true clinical science. It is obvious that in a charitable institution where accommodation is limited and many patients are forced to wait weeks and even months for admission, the reduction of the number of surgical complications, such as sepsis, phlebitis, cystitis, etc., is one of the easiest ways of economising hospital funds and making the hospital service of the greatest benefit to the greatest number. An index of efficiency could be obtained by multiplying every patient-day lost in a charitable hospital by the daily *per capita* expense.

### How the System Works.

To effect improvement the first step would be, as in the case of the author's experiment, to admit and record the lack of perfection, the next to analyse the causes of failure and ascertain to what extent these causes are controllable. The reader must suppose himself the chief of a surgical service or a member of a hospital board. The "End Result" cards of the week are before him; a service of fifty to a hundred beds could in this manner be reviewed in an hour weekly. He must read them through, pass those which he regards as satisfactory, and, either in consultation or from his own knowledge of the individual case, annotate upon the rest the probable cause of failure.

Such a system, if conducted by a chief endowed with powers of leadership and the gift of stimulating enthusiasm in his subordinates, could not fail to have a considerable effect in exciting a spirit of friendly emulation and *esprit de corps* throughout the whole working staff. Judicious praise should leaven the necessary criticisms and suggestions which it would be his duty to make, for to the conscientious worker nothing is so deadening as to be ignored.

### The Surgeon's Failures an Asset.

As we have previously remarked, under existing conditions it is the duty of no one person or committee to make such strictures. It is, as anyone with clinical experience knows, no uncommon thing to find patients, suffering from some incurable or undiagnosed condition, occupying for months at a stretch the all-too-limited accommodation of our large city hospitals. In some, no doubt, the plea may be made that although nothing effective is

* *A Study in Hospital Efficiency.* By E. A. Codman, M.D.

THE FINANCE OF SURGERY—*(continued)*.

being done to bring about a cure, yet they are useful from a teaching point of view as clinical material. And here we come upon the second of Dr. Codman's arguments in favour of the system he advocates. A hospital appointment is a big asset to the doctor, and especially to the surgeon. The faith of the general public in any particular physician or surgeon depends scarcely at all upon the quality of the work he does in his capacity as hospital operator. His failures are seldom heard of, and his successes appear with due *réclame* in the columns of the medical press. If a surgeon did no cases which were unsuccessful the demand for his services would fall, and he would soon be unable to make a living. By means of the experience gained at the expense of his non-paying patients, he is enabled to perform similar operations in private with greatly reduced risks. Further, the invaluable practice, that "keeping the hand in," wherein lies the essence of the surgeon's skill, is only rendered possible by an abundance of human material upon which to maintain and perfect his technique. Even the trustees of such institutions seem ignorant of this "bread-and-butter" point of view of their appointments, and feign to regard the services given by members of the staff as gratuitous. Nevertheless it is common knowledge that a surgeon without the *kudos* of a teaching or operative post at a large municipal hospital has little prospect of making headway as a specialist. The author was, at one time, a surgeon on the staff of the Massachusetts General Hospital. Reasons not unconnected, we may assume, with professional jealousy led to his resignation after he had founded the private home which we are now considering. He then found himself, from the financial point of view, in direct competition not only with the hospital from which he had lately resigned, whose charge to paying patients varied from 28 to 42 dollars a week—the fee for operation being in all cases additional—but with other private homes whose surgeon in charge, as the holder of a public appointment, was enabled to demand fees of from 300 to 1,000 dollars per operation. There were, moreover, a large number of men—the author himself estimates them at 1,000—in the State of Massachusetts who occasionally practise surgery, and who he maintains have too few opportunities for doing so to justify their undertaking any. Against this formidable array he could offer accommodation fully equal, he claims, to that usually obtained in a 25-dollar-a-week hotel at a similar price. The undertaking was, therefore, a strictly commercial venture, and, as such, could be deemed successful only by a strict consideration of profit and loss; but a commercial venture could at least bring its wares into publicity by the accepted channels. Medical professionalism, too, has its methods of advertisement, but they are devious. A surgeon can bring his name into prominence in several ways:—

(1) By the recommendations of patients and friends, legitimate if scarcely unbiassed judges.

(2) By his position on a hospital staff.

(3) By the pseudo-scientific discussion of topics of popular interest in the lay press.

(4) By delivering lectures on specialist topics to his medical brethren, and by the extravagant exploitation of new methods and discoveries.

(5) By the publication in the technical press of series of successful cases, and his publisher's booming of works written on his particular subject.

Commercialism v. Professionalism.

It would be a matter of endless controversy to decide to what extent these methods are justifiable from the standpoint of strict professionalism. Dr. Codman's contention is nevertheless readily appreciable by the fact that he is no longer the holder of a hospital appointment. Any reputation he may build up is entirely at the expense of his commercially profitable *clientèle*. Moreover, without such prestige his pronouncements on medical and surgical questions would carry little weight with his professional colleagues. In order to render his home a success, he is forced to devote so much time to it that he has few opportunities of exchanging views with his fellow-workers. Lastly, he is, as he tersely puts it, forced to advertise his wares by the publication of a pamphlet setting forth the nature of the results obtained by him instead of having his name passed on by an ever-changing procession of students, nurses, patients, and doctors at the hospital, where nobody knows enough to criticise results or dares to compare them with those of other colleagues.

It would seem, then, that, situated in the somewhat anomalous position of a specialist whose prospects of success depend solely upon the results obtained upon the very class of patient from whom he gains his livelihood, any form of advertisement which relies upon the demonstrated power to "deliver the goods" is a perfectly legitimate means of augmenting income. The spirit of rigid professionalism in its primary sense should imply that the best man reaches the top in virtue of his merits, unaided by baser forms of publicity; but as the author has tried to show, and as we have previously demonstrated in these columns, to secure a hospital post does not of necessity prove its possessor to be endowed with the manifold qualities required in the make-up of a scientific surgeon or physician. Nepotism, social qualities, an income independent of the exigencies of professional life, are all factors which tend to prevent the best man being selected for any vacancy. The adoption of the "End Result" system, which Dr. Codman so ably advocates, would serve as a double safeguard to hospital efficiency. By publishing annual data of every case treated in public and private institutions alike, the lay public would learn to distinguish readily between those practitioners whose reputation was based upon a sure foundation of skill and knowledge, and those whose claims have no such basis. Moreover, within the institution itself a weekly inspection of "End Result" cards would reveal to the administration many minor faults in surgical technique and nursing which might otherwise pass unheeded.

